# A Novel Approach about Edible Packaging Materials Based on Oilcakes—A Review

**DOI:** 10.3390/polym15163431

**Published:** 2023-08-17

**Authors:** Ancuţa Petraru, Sonia Amariei

**Affiliations:** Faculty of Food Engineering, Stefan cel Mare University of Suceava, 720229 Suceava, Romania; sonia@usm.ro

**Keywords:** valorization, by-products, bioactive compounds, edible films, biopolymers

## Abstract

Due to the growing global population and subsequent environment degradation, as well as changes in the climate, changing consumers’ dietary habits is necessary to create strategies for the most efficient use of natural resources to eliminate waste in the food supply chain. The packaging of food is essential to preserve the food’s properties, extend its shelf life and offer nutritional information. Food products are packaged in various materials of which the most used are plastics, but they have a negative impact on the environment. Various efforts have been made to address this situation, but unfortunately, this includes recycling rather than replacing them with sustainable solutions. There is a trend toward edible packaging materials with more additional functions (antioxidant, antimicrobial and nutritional properties). Edible packaging is also a sustainable solution to avoid food waste and environment pollution. Oilcakes are the principal by-products obtained from the oil extraction process. These by-products are currently underused as animal feed, landfilling or compost. Because they contain large amounts of valuable compounds and are low-cost ingredients, they can be used to produce materials suitable for food packaging. This review covers the recent developments in oilcake-based packaging materials. Special emphasis is placed on the study of materials and technologies that can be used to make edible film in order to research the most suitable ways of developing oilcake-based film that can be consumed simultaneously with the product. These types of materials do not exist on the market.

## 1. Introduction

Food packaging plays an important role in the food supply chain. An adequate packaging can reduce wastes and guarantee food quality during storage. The most frequently used materials are plastics due to their reduced cost, easiness of shape and weightless character, but unfortunately, they are affecting the environment [1]. To address this situation, various efforts and actions have been made by governments, companies and researchers [2]. The companies collect and recycle these materials rather than replace them with sustainable solutions [3]. Moreover, agreements between the companies and researcher centers must be reached in order to achieve the best solutions faster [4,5]. There is a high expectation for natural, eco-friendly, biodegradable and edible materials that can improve food safety and quality (by adding substances that enhance antioxidant and antimicrobial properties) [6].

The difference with the conventional packaging is that this is integrated with the food product so they can be consumed together without having to throw away the packaging.

Every year, about 1/3 of the food produced is wasted or lost [7]. A possibility to reduce these losses is by their valorization through different strategies. Conventional management methods include landfilling, composting, thermal treatment and animal feeding. However, to obtain a more integral management, the emerging technologies such as the extraction of bioactive compounds and production of edible films must be taken into account [8].

Oilseeds are grains used primarily as a source of vegetable oil. After oil extraction, valuable by-products called cake or meal remain. Due to their composition, which is rich in protein and polysaccharides, they are promising ingredients for eco-friendly, edible biopolymers that could be used as packaging materials.

This review aims to collect the information available about the edible packaging system and the incorporation of the by-products resulting from cold oil extraction in these systems. The studies found in the specialized literature highlight the possibility of using the proteins extracted from oil seed flour in the production of biodegradable films. From the literature study, the packaging obtained with proteins extracted from the oil industry by-products have good gas barriers. Their mechanical, physicochemical, thermal, water vapor barrier, and water sensitivity properties can be improved with different optimization processes.

The novelty of this review is to study if they can be incorporated entirely in films that can be consumed simultaneously with food products. In addition, it gives them a higher nutritional and organoleptic value. 

## 2. Oilcakes, General Aspects and Possible Valorization Methods

Oilseed crops are grown mainly for oil production. Oilseeds have an important role in providing balanced diets rich in fibers, antioxidants, vitamins, minerals and fatty acids. Globally, the most cultivated oilseeds are soybean, rapeseed, cotton, sunflower and groundnut. Other seeds with relevance in the food sector due to their pharmaceutical properties are sesame and hemp [9]. 

The extraction of oil from oilseeds produces valuable by-products (cake/meals). The process of extraction is completed either by using solvents, hot (100 °C)/cold (50–60 °C) pressing or modern/green technologies (pulsed electric field, high voltage electrical discharges, pressurized liquid and high hydrostatic pressure) [10]. 

There are two types of oilcakes, namely edible and non-edible. The edible oilcakes have high nutritional value due to their significant amount of residual oil, proteins, minerals and vitamins. Due to their content in toxic compounds, the non-edible oilcakes are used as manure [11]. 

The composition of oilcakes is presented in Table 1 and varies depending on quality of seeds/nuts, condition of growing of the raw material, method of extraction and storage properties [12,13]. Table 1 indicates that despite the oil extraction process, the oilcakes contain high amounts of oil, especially sesame. On the other hand, hemp and sunflower oilcakes are rich in fibers. All the oilcakes have a high concentration of proteins varying from a minimum of 10.30% to 53.98%.

Unfortunately, the cakes can contain undesirable antinutritional ingredients (sinapine, phytic acid, tannis, saponins, glucosinolates, cyanogenic glycosides, chlorogenic acid and trypsin inhibitor [64,65,66,67,68]) which must be removed because they can limit their future utilizations. By enzymatic, fermentative, chemical (pH modification, extraction of proteins, ammonification or the addition of sodium carbonate, choline, methionine or ferrous sulfate) and physical (heat-cooking, dehulling, toasting, autoclaving) methods [11,35,69,70,71,72,73,74,75], these undesirable substances can be eliminated/inactivated. 

**Figure 1:**
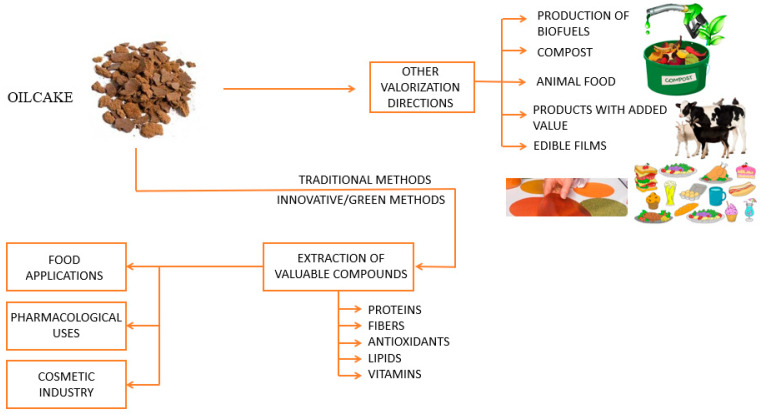
Different valorization strategies for oilcake.

The possible applications of the oilcakes are as energy sources, landfills, animal feeds, substrate for surfactants/antibiotics/vitamins/enzymes, extraction of bioactive compounds and the development of value-added food products or biopolymers packaging materials (Figure 1) [10,11,76,77,78,79,80,81,82,83].

## 3. Edible Packaging

The packaging offers food products protection against potential physical damage and chemical and/or biological contamination (delaying food spoilage, maintaining food quality and safety, and extending shelf life). The essential functions of packaging are covering, preserving, maintaining safety and quality, increasing the shelf life of food products during transport, storage and marketing. Packaging also provides information on the nutritional value and end use of food products [84].

The use of non-biodegradable and non-renewable materials in packaging has led to numerous environmental concerns regarding their decomposition and the overexploitation of natural resources. As a result, suitable alternatives are sought to minimize the use of traditional materials [85].

As a definition, edible packaging consists of materials used for encapsulating different foods to preserve the properties; in addition, they can be consumed with food. There are two forms of edible packaging: namely, films and wrappers/coatings. They fulfill the same role, but they are different concepts (the main difference is their physical appearance). Coatings are applied directly to the surface of products (by dipping, spraying or brushing); instead of films, they can be used as a solid layer to cover food products (thus preventing the transfer of moisture, oxygen and soluble substances) [86]. 

Consumable films can be made by two methods—wet and dry processing. Wet processing is the most common method, and it is also called solvent casting. The film-forming macromolecules were dissolved/dispersed in a suitable medium (water, alcohol, mixture of different solvents), and by its evaporation, the film is formed. In contrast, the dry method involves thermo-mechanical processing such as compression molding (extrusion) [13].

The casting is widely used in research and can be completed through equipment that exists in the laboratory. Unfortunately, in the wet process, solvent removal is a time-consuming and energy-expensive step (due to the price associated with drying oven maintenance). On the other hand, extrusion is a mass scale, faster, and more energy-efficient process that lowers the production cost of biopolymers to compete with synthetic polymers [87].

There are several undeniable advantages of these materials [6,88]:

- Edible and biodegradable nature;
- The amount of solid waste is considerably reduced;
- The organoleptic properties of food are improved;
- The nutritional properties are improved by adding adjuvants;
- Brings the possibility of packing items individually;
- Antimicrobial properties;
- The possibility of using a range of by-products (e.g., agricultural waste) from different activities.

## 4. Technological Properties of Edible Films/Coatings

Coatings and edibles are used in film in the same way as any other packaging to preserve the properties of the original food and must present some specific characteristics [89]:

- The ingredients included in the composition of the films must be safe (generally recognized as safe (GRAS)). The main goal is to avoid toxic and/or allergenic components;
- Adequate mechanical properties to prevent food surface damage during handling;
- Adheres to food surface;
- The film has a pleasant taste or is tasteless;
- Stability over time and above all avoiding the development of molds;
- Reducing the dehydration of the wrapped product;
- Maintaining adequate gas transfer, especially for oxygen and carbon dioxide;
- Avoid losing the components that are responsible for flavor and nutritional value;
- Improvement of structural properties;
- The overall presentation of the final product, the realization of some classic performances of the package from the point of view of design. Otherwise, the product may be rejected by consumers;
- Low costs—to justify a major shift in the food industry ideology, costs must be lower;
- Manufacturing processes must be easy and economically viable. Maintenance and cleaning of the devices used must be easy.

### 4.1. Mechanical Properties

The mechanical properties are important because the packaging must greatly ensure the integrity (protection against mechanical shocks) of the packaged food products from harvest, processing, and storage to consumption. These include tensile strength (TS), elongation at break (EB), deformability and elastic modulus (E). TS is expressed as the maximum stress/load that a material can withstand, and EB is the material’s elongation to break [13,87].

The mechanical properties of edible packaging depend on the nature and composition of the film-forming solution (the structure of the polymer chains, the coherence of the polymer matrix, the interaction between the additive and the matrix), the way the film is produced and the presence of plasticizers (they can improve elasticity). In addition, films based on biopolymers are significantly influenced by the water content of food as well as the humidity and temperature of the environment; therefore, if their variations occur, their physical properties can be modified, which limits their use. For example, hydrophilic films at higher moisture levels absorb water more easily, increasing its plasticizing effect, thus reducing tensile strength and increasing extensibility [84,90].

### 4.2. Barrier Properties

Mass transfer between packaged food, packaging material and atmosphere is important in evaluating future packaging applications. The edible films exhibited barrier properties. The packaging provides safety against chemical changes, ensuring the quality and the shelf life [91].

The most important barrier properties are permeability to gases, water vapor, light, oil and aroma. The migration of oil, flavor or smell, the permeability of oxygen, carbon dioxide and other gases as well as the release of constituent materials of the packaging to the food could affect their quality [13]. 

The effectiveness of the barrier properties of the packaging materials is achieved by permeability measurements. Permeability describes the rate at which gases (or vapors) are transferred through the packaging material. This process is influenced by the pressure variation between the two surfaces of the material at humidity and temperature stability conditions [92]. The barrier properties depend on the chemical composition of the film, the process of obtaining the film and the method of applying the film to the product’s surface [13,93].

#### 4.2.1. Affinity for Water

Water sensitivity can be evaluated by the film’s moisture content under specific environmental conditions, water absorption capacity, and water solubility [13].

Water solubility is of major importance because it conditions the use of films in technological applications. A high solubility is desired in the case of foils/coverings that are consumed simultaneously with food [94].

Edible packaging can be used to inhibit the exchange of moisture between finished food products and atmospheres. Water activity (aw) is an important factor affecting the sensory quality and shelf life of foods. The parameter is determined based on moisture levels and interactions between water molecules and the other ingredients. Some chemical and enzymatic degradation processes, microorganism development and textural properties depend on a_w_ [95].

Water vapor permeability (WVP) is a parameter that directly affects the freshness of the packaged product. Although biopolymer films are a good barrier to gases, they are a poor barrier to water vapor. Their hydrophilic nature is one of the limiting factors of their widespread use. WVP can be reduced by adding lipid components (wax or essential oils), which increase hydrophobicity, or mixing with other biopolymers [96].

#### 4.2.2. Gas Permeability (Oxygen (OP), Carbon Dioxide (CP))

Oxygen can cause food spoilage reactions (fat and oil rancidity, enzymatic degradation and loss of vitamins) and the growth of microorganisms. Other gases that play a relevant role in food packaging include carbon dioxide, which can be added to packaging for various purposes. It is used to suppress or limit products such as microbial growth on fresh meat, cheese or bakery products or to reduce the rate of food respiration [91].

The packaging of fresh fruit and fresh vegetables requires films/coatings with low OP (too low to produce anaerobic conditions favorable for the formation of flavor substances and ethanol production) and high CP because it is necessary for respiration [97].

Gas permeability can be influenced by relative humidity (RH), temperature, thickness and WVP. Maintaining RH is crucial for maximizing the effectiveness of gas barrier properties, as a higher RH can substantially increase OP. In addition, thicker shells limit gas exchange [87].

### 4.3. General Appearance and Optical Properties

The aesthetic aspect of a food package contributes to the consumer’s decision to purchase the products. Therefore, the optical properties (color, transparency and ultraviolet barrier properties for ultraviolet and visible radiation) of the material are essential features for films.

Sensory and nutritional qualities of food products can be modified by exposure to light. The UV and visible barrier properties can be assessed using a spectrophotometer (exposure of films to wavelengths between 200 and 800 nm). Packaging materials must have low transmission for UV radiation (which increases the shelf life of packaged food) and high transparency in the visible region (to give consumers visual control) [98].

Polymers from natural resources generally include colored organic molecules. Colors can be quantified using three distinct values in a three-dimensional space. Preferably, the materials should be transparent, odorless and tasteless. Hydrocolloid materials are substantially more neutral than lipid and wax-based materials [87].

Edible coatings and films improve the appearance (imparting gloss, color), sensory attributes and texture (surface smoothing, non-greasy/tacky surface). At the same time, there are psychological limitations on the part of consumers regarding the consumption of the food product simultaneously with the films or coatings [95].

## 5. Food Packaging Laws and Regulations

There are laws that regulate the quality control of packaging in terms of the interaction with food products (packaging–product relationship). These regulations are complex due to the diversity of materials used (paper, glass and plastic), their presentation (boxes or bags) and the characteristics of the food products (moisture, fat or alcohol content, pH and freshness). Packaging must meet five basic requirements to be commercially available: (i) it should not present any risk to human health, (ii) it should not change the physico-chemical composition of the food, (iii) it should not change the organoleptic characteristics of the food, (iv) it should be manufactured and treated in accordance with good manufacturing practices and (v) it must not present misleading information about the product contained [99].

Legislation established by the International Organization for Standardization (ISO) also deals with regulations involving the production, distribution and use of packaging materials (ISO 18604:2013 [100]). There are laws that regulate the production of packaging waste and laws that have restricted the use of waste that directly or indirectly leads to the pollution of flora and fauna. However, the total elimination of packaging is impossible, because food products always need protection to allow them to be preserved until consumption. For this reason, edible films and coatings have become a promising alternative for the preservation and clarity of food quality during processing and storage [93].

In Europe, edible packaging materials are included in Regulations EC 1331/2008 and EU 234/2011 for food additives, enzymes and flavorings. Raw materials used for packaging development must be part of this list and comply with legislation/authorizations. To achieve GRAS status, the manufacturer must apply for Food and Drug Administration (FDA) approval [77]. 

## 6. Materials Used in the Preparation of Edible Films 

Edible films and coatings are materials containing the main biopolymers recognized as safe (GRAS test). Proteins (of animal or vegetable origin), polysaccharides, lipids and combinations thereof are used to develop edible films and coatings. Films based on polysaccharides and proteins provide hydrophilic character, while those based on lipids offer exceptional barrier properties. Out of them all, the most attractive options are proteins because they also offer nutritional value [101].

### 6.1. Proteins

Proteins most commonly of plant origin include those from corn, wheat gluten, soy proteins, oilseeds or cereals, and those of animal origin include collagen, gelatin, casein, keratin, proteins from egg white and whey [86]. 

Protein-based films have optimal barrier (against oxygen, carbon dioxide and aromas), mechanical and optical properties due to their structured, ordered, and compact hydrogen network [102]. Unfortunately, these films are susceptible to moisture due to their hydrophilic nature. Thus, in order to limit the water absorption capacity, it is possible to resort to the incorporation of hydrophobic components or their intercalation between two hydrophobic polymer layers. Additional methods include the addition of cross-linking aid, use of biopolymer mixture, nanoparticle reinforcement, lamination, irradiation, ultrasound, and microfluidization. Both wet and dry methods can be used to create the protein film [103]. 

Moreover, due to the fact that the packaging based on proteins has the ability to inhibit the penetration of oxygen, it can be used for high-fat products to inhibit the lipid oxidation, which is the major cause of deterioration in quality and shelf life [104]. Films made with zein, gluten, soy protein and whey allow oxygen to penetrate more than collagen films [105].

Collagen-based films are obtained by extrusion and have good mechanical properties (TS) [106]. The development of films with gelatin requires a wet process and the presence of poor mechanical and barrier properties due to its hydrophilic nature [91]. Wheat gluten films were obtained by intensive extrusion, which was followed by compression and casting. The clarity of the films depends on the purity of the gluten mass and the medium. The films have excellent mechanical and barrier characteristics (oxygen, carbon dioxide and lipids) as well as thermal stability and low water resistance [107,108].

Soy protein films are fine, transparent and flexible. They showed good oxygen barrier properties at low humidity conditions, but the major disadvantages are the low mechanical strength and lack of thermal stability [109] (the latter can be improved with triethanolamine [110] and sodium dodecyl sulfate [111], while the increasing of protein concentration and moisture content reduced heat stability [112]).

Whey proteins are preferred over total milk proteins as the latter produce crystallization in the presence of lactose; in addition, they have exceptional functional and film-forming characteristics [113]. The films exhibit excellent transparency, flexibility, and good barrier properties to gases, flavors, and lipids, while the barrier ability against water is poor. The latter can be completed by incorporating essential oils [114].

### 6.2. Polysaccharides

Polysaccharides are the most abundant natural polymers. Polysaccharides used in the preparation of edible packaging are cellulose, hemicellulose, starch, pectin, gums, alginate, chitosan and fibers. Polysaccharide films present an ordered network of hydrogen bonds, which makes them effective for blocking oxygen. However, they are less effective as a barrier against water [101]. Despite this shortcoming, polysaccharides can be used to protect foods from oxidation. Polysaccharide compounds in edible films and coatings contribute to the following physical properties: hardness, fracturability, compactness and viscosity [115].

Cellulose-based films prevent oil absorption and are therefore used for confectionery [116]. Starch consists of amylose and amylopectin. Among them, amylose is generally used for film formation due to its low oxygen permeability, heat resistance, flexibility and water solubility. However, it presents a poor barrier to water vapor [117,118].

Pectin is widely used for film, making chemical use of its biocompatibility, biocompatibility, versatile properties, and physical properties, such as selective gas permeability [119].

Alginate is isolated from brown algae. Its colloidal nature including thickening and stabilizing properties makes it a competitive material for edible films. The food film based on alginate has little resistance to moisture or water due to the hydrophilic nature of alginates [120]. Chitosan films exhibit oxygen and carbon dioxide barrier properties as well as antimicrobial properties [121].

### 6.3. Lipids

Lipids can be mixed with other biopolymers to obtain films with good barrier properties (retard gas diffusion) and low water absorption capacity. Unlike proteins and polysaccharides, lipids alone cannot form an edible film. The main disadvantage of the lipid film is its fragile nature. It also imparts a waxy, greasy texture and taste that is not desirable for packaging material. The improvement of vapor barrier and functional properties (antibacterial and antioxidant) can be achieved by incorporating essential oils [101]. At the same time, for films with reduced mechanical strength and increased permeability to oxygen, the combination with a hydrophilic material or lamination with another lipid layer can improve these aspects [122].

### 6.4. Plasticizers

Plasticizers are low molecular weight compounds that combine with base biopolymers to increase their thermoplasticity. The main role of these compounds is to decrease the polymer–polymer interactions, which increases the free volume and movement between the polymer chains. These properties lead to the lowering of the glass transition temperature. Most plasticizers are highly hydrophilic and hygroscopic to attract water molecules and form a hydrodynamic complex [123].

There are two types of plasticizers: internal and external. Internal plasticizers enter the composition of polymer molecules. They increase the free volume (provides more space for the polymers to move) and flexibility by lowering the glass transition temperature (Tg), thus lowering the elastic modulus. External plasticizers are substances with low volatility; they interact with polymer chains but are not chemically attached to them by chemical bonds [124].

The most important characteristics of plasticizers are their compatibility, efficiency and permanence. The plasticizer must be compatible with the polymer system in both the processing and use temperature range; they must be harmless and odorless. Efficiency is achieved if the plasticizer fulfills the role at lower concentration and has a high diffusion capacity in the polymer matrix. The permanence of plasticizers refers to their tendency to remain in the material. This depends on the size of the molecule (the larger it is, the lower the volatility, and implicitly, the higher the permanence), the diffusion rate (unfortunately, a high diffusion rate ensures at the same time high efficiency but also a lower permanence), volatility, stability to water and resistance to solvents and oils. Therefore, the plasticizer should have a low vapor resistance and a low diffusion rate in the polymer [125].

The addition of plasticizers in edible packaging gives flexibility, decreases brittleness, increases their hardness and prevents the appearance of cracks and pores [126]. Plasticizers generally lead to superior mechanical properties, but due to their high hygroscopic properties, they increase the WVP and decrease gas, moisture, and flavor compound-blocking properties [97].

Plasticizers are required in proportions from 10% to 65% depending on the stiffness of the polymer. They improve the polymer formation process and can be used at higher temperatures. The main plasticizers applied in films and coatings are water, glycerol, propylene glycol, sorbitol, polyethylene glycol, xylitol, mannitol and corn syrup [89].

### 6.5. Additives

Edible films and coatings can serve as carriers for numerous active compounds such as antioxidants and antimicrobials, flavors and colors that maintain the quality, safety and shelf life of the packaged product. Their incorporation is only possible up to a certain level where they start to influence the physical and mechanical as well as visual properties of the films [127].

### 6.6. Surfactants/Emulsifiers

Edible films and coatings can serve as carriers for numerous active compounds such as antioxidants and antimicrobials, flavors and colors that maintain the quality, safety and shelf life of the packaged product.

Surfactants are ionic, non-ionic and amphoteric macromolecular stabilizers that can lower the surface tension between two immiscible phases. The major function is to prevent phase separation by maintaining the balance between the hydrophilic and lipophilic phases [128].

The hydrophilic–lipophilic balance (HLB) shows the attraction of surfactants to water or oil. This can be calculated with the Davies Equation (1):

HLB = 7 + (sum of hydrophilic groups) + (sum of lipophilic groups)(1)

The higher the HLB values, the higher the attraction for water, and the lower the values, the higher the attraction for oil [129].

Emulsifiers commonly used in edible films/coatings include the following: glycerol monostearate, sucrose stearate, soy lecithin, sodium dodecyl sulfate, silvered ethyl lauroyl hydrochloride, sorbitan laurate 20 and 80 and polysorbate 20 and 80. Surfactants produce films’ properties, lower the WVP and improve the barrier properties. In addition to allowing adhesion between the coating and the final product, it prevents the formation of rough surfaces and leads to the formation of uniform edible films and coatings [130].

Lecithins are the most important emulsifiers; they are mixtures or fractions of phospholipids of vegetable origin. Their introduction into edible packaging affects the color (they become more opaque), solubility, barrier properties (decreased WVP), mechanical properties (they are flexible, decrease TS and increase E) and their microstructure [97].

## 7. Development of Edible Films and Coatings Using By-Products Resulting from the Extraction of Oil from Oilseeds

**Figure 2:**
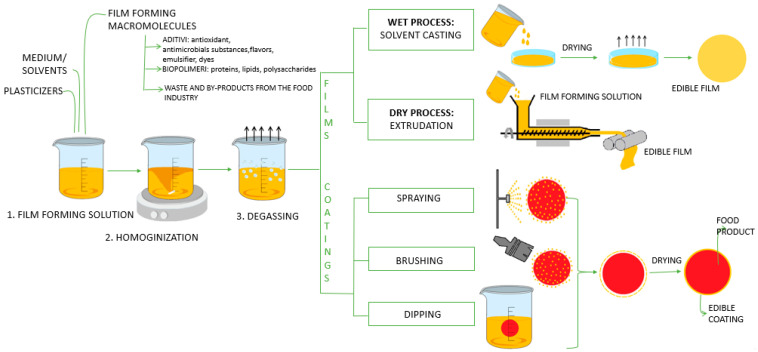
Schematic presentation of the development of food packaging.

The packaging industry is still evolving depending on consumer lifestyle changes, market changes and the need to limit the impact of food waste. The schematic presentation of food packaging development is shown in Figure 2.

The use of residues and by-products from the food industry has aroused great interest in the production of edible packaging. The recovery of by-products promotes the notion of sustainability and their recycling/reuse, thus adding more value to food and reducing also the costs and risks of their disposal in the environment [86].

An interesting approach is the usage of whole oilcakes, which are naturally complex combinations of protein, polysaccharides and lipids. Unfortunately, the application of oilcakes as raw materials for film production is limited due to the presence of antinutritional factors. Thus, the incorporation can be achieved by extracting the proteins or by cooking the residues. Sunil et al. [12] investigated the potential of using raw or cooked oilseed cake in food application. The cooked residues retained the nutrients, and are moreover, they are more sensorial acceptable than raw materials. Moreover, the cellulose components strengthen the lipid barrier of the obtained films.

Commercially, the most popular packaging material is low-density polyethylene (LDPE) due to its mechanical (good tensile and impact resistance) and barrier (moisture and WVP) properties, but it has a low barrier to oxygen [131]. Similar characteristics can be obtained when making edible packaging with proteins extracted from oilcake. From the studies presented in Table 2, we discovered that they have good barrier properties against oxygen, nitrogen and carbon dioxide (for example, the gas properties of pumpkin oilcakes-based films are 150–250 times better than the commercially polyethylene and polypropylene) and low permeability to moisture (the water vapor permeability of sunflower protein isolate films was similar to that of low-density polyethylene films). The last properties make them ideal for hydrophilic surface (meat and dairy products). When it is desired to cover products that require protection against moisture, lipids can be incorporated.

Compared to conventional polymeric films, the protein oilcake-based films presented weak mechanical properties that can be improved by the addition of essential oil [132] or crosslinking with different concentrations of malic, citric and succinic acids [9]. On the other hand, films with protein isolated from pumpkin oilcake showed similar or higher EB than that of cellophane, about 20%, but lower than polyethylene, between 300% and 500% [133].

Another characteristic of these films is their stiff and brittle structure than can be improved with the addition of a plasticizer. The addition, however, affects the barrier properties (gases, aroma, moisture, antioxidants and oil). It is necessary to optimize the experimental conditions (the ingredients and their proportion, pH and temperature) in order to obtain films with best properties.

The incorporation of oilcakes in films affects also the optical properties (films are opaque), which is beneficial to protect food against chemical degradation. The difference in color also differs on the type of oilcakes used.

The films achieved so far were primarily developed using the solvent casting method at laboratory scale where the film-forming macromolecules are dissolved in an appropriate medium and plasticizer, and the evaporation of these leads to the film. Their production at industry scale is delayed due to their barrier and mechanical properties limitation as well as their high processing cost.

### 7.1. Methodology

For our research, a systematic search on international databases (Google Scholar, MDPI, SpringerLink, Science Direct and PubMed) was conducted. The research was based on specific keywords such as edible packaging, oilcakes, oilseeds, film properties, agroindustrial waste, circular economy, environment pollution, and edible packaging materials. No timeline for the literature research was applied so as to present all the results obtained in the field up to the present moment. The research includes review articles, short communications, conference materials and book chapters.

### 7.2. Consumer Acceptance

The consumers accept the edible packaging if they consider it safe. Although the idea is that these packaging materials can be eaten along with the food, limited studies have been conducted to verify their biodegradibility and edibility. The acceptance of edible films can be limited by lack of awareness and fear. Marketing strategies (awareness programs about carbon footprint and waste, price discounts, attractive offers and advertisements) might be helpful to attract consumers. Another reason for their lack of acceptance is the fear for the presence of allergens that can be resolved by a proper labeling. The last factor includes the cost; therefore, the edible films have a price 10–50 times higher than the petroleum-derived plastic films. In order to attract consumers, the cost should be lower or equal to the petroleum derived [134,135].

**Table 2:** Literature studies on the incorporation of flakes into edible films.

Composition of Films and Coatings	Effects	References
**MUSTARD OILCAKE (MOC)**		
MOC defatted flour (14 g), glycerol (2 g).	- the film delayed the growth of Listeria monocytogenes in smoked salmon stored at 5 °C, 10 °C and 15 °C; - more visible effect when the coating was applied before inoculation than when it was applied after inoculation.	[136]
MOC defatted flour (11.9–14 g), xanthan (0–15%), glycerol (2 g), polysorbate 20 (1%).	- films with 5% xanthan demonstrated antioxidant properties; coating salmon with these films established its stability against lipid oxidation without conferring a negative sensory quality.	[137]
**SOYBEAN OILCAKE (SYOC)**		
Defatted flour from SYOC (26–59%), xanthan (10–90%), glycerol (0–16%), lacto-peroxidase (0.1–0.7%).	- the films with lactoperoxidase inhibited Salmonella Typhimurium; - increasing the concentration of xanthan increased the strength of the film, and increasing the amount of glycerol increased the elasticity and reduced the WVP.	[138]
Protein isolates from SYOC (8%), glycerol (2.4%), essential oil of cinnamon and ginger (0.025–0.100 g).	- films with cinnamon essential oil were more resistant and elastic than those with ginger essential oil; - the WVP did not present any changes; - the cinnamon oil of the product changes the optical properties more significantly than the ginger essential oil.	[139]
SYOC or protein isolates from SYOC, glycerol (70:30), polylactic acid (PLA, 0–50%), sugar cane bagasse (0–15%).	- the tensile strength of the material based on protein isolates was higher, while the impact resistance was higher in the material based on SDSO; - the material based on SDSO showed a lower percentage of water absorption; - further addition of these materials was made by the addition of PLA (40%) and/or bagasse from sugar cane (15%).	[140]
2 different films were made: 1. a film with low-density polyethylene (LDPE) and addition of 20% SYOC; 2. multilayer film with SYOC in the middle of two layers of LDPE.	- SDSO increased resistance and oxygen barrier properties, while LDPE improved water and water vapor resistance; - the oxygen transmission rate in the multilayer films decreased by 38% due to the presence of soy flour; - the elongation at break of the films containing soy decreased by up to 14%, and no trend was revealed for the tensile strength.	[141]
Protein isolate from SYOC (5%), glycerol (50%), oilseed meal (5%, hempseed, flaxseed, pumpkin, sesame, sunflower), glycerol (50%).	- the oilcakes flours increased the opacity of the films, the color of the films varies depending on the type of oilcake used. The highest proportion of green color was observed for the sunflower flour film; - all films with meal showed lower TS than those with only soy protein isolates. TS values vary according to the following scheme: sesame < pumpkin < hemp < sunflower < flaxseed; - the values for water vapor permeability follow the following scheme: sesame > pumpkin > hemp > flax > sunflower; - the microstructure was affected by the addition of sawdust, the homogeneous and smooth structure became rough; - the humidity increased due to the presence of glycerol as well as the chemical properties of the shavings (they have hydrophilic components with water retention capacity); - the solubility decreased due to the presence of fat from oilseeds (pumpkin < flaxseed < sunflower < hemp < sesame).	[142]
**HEMPSEED OILCAKE (HSOC)**		
Protein concentrates from HOC (200–400 mg), glycerol (10–50%), microbial transglutaminase (mTG, 0–40 U/g protein concentrates).	- the mTG treatment of the film product is more homogeneous, fine, resistant, and flexible, with a high permeability capacity against gas and a low permeability capacity against water vapor; - the packaging is suitable for packing fresh fruit (apricots and dates) because it allows them to breathe, and at the same time, they are the same color and do not negatively influence the acceptability on the part of the customer.	[143]
**PUMPKIN OILCAKE (POC)**		
Protein isolates from POC (10%), glycerin (0.3–0.6 g).	- the pH and concentration of the plasticizer affect the mechanical properties and solubility of the films; - the films with 0.4 g of glycerol demonstrated excellent barrier properties for oxygen, nitrogen and carbon dioxide (150–250 times better than synthetic polymer films); - the films made at pH = 10–12 demonstrated the best properties.	[144]
POC (40–95%), gelatin (5–60%), glycerol (0.1–0.2 g).	- the best resistance was obtained for films with 60% gelatin, 40% sawdust, 0.15 and 0.2 g glycerol; - the elongation at break increased by increasing the addition of sawdust; when glycerol was introduced, it increased two to three times more compared to the 100% gelatin film.	[145]
POC (10%), glycerol (30%), the film was cast on a polyethylene (PE) film to obtain a two-layer film.	- the material benefited from the different nature of the two layers: good oxygen barrier properties for water vapor and light (due to the PE layer); water sensitivity and improved mechanical properties (due to the layer based on biodegradable material); - the material is suitable for packing a wide range of food and other sensitive products, but you must store in dry conditions, avoiding direct contact with water.	[146]
POC (10%), glycerol (30%), guar–xanthan gum (0.2%), thyme or basil essential oil (3, 4, 5%).	- the incorporation of essential oils increased film thickness, improved barrier properties (against water vapor and light) and significantly (*p* < 0.05) reduced sensitivity to moisture/water.	[147]
Double-layer film: 1. POC (10%), glycerol (30–50%), guar–xanthan gum (0.1–0.5%); 2. 10% mixture of zein in 85% ethanol and polyethylene glycol (PEG400).	- the film with the best mechanical properties was obtained with the lowest concentration of glycerol (30%) and the highest concentration of guar–xanthan (0.5%); - the speed of transmission of water vapor and carbon dioxide through the film increased by increasing the concentration of both additives; - the presence of an increased amount of carbon dioxide in the packaging ensures the quality of the food products and extends the shelf life, which is why the films lend themselves to the packaging of cheeses, fruits and fresh vegetables; - all films showed good barrier properties against oxygen.	[148]
3 membranes were made: 1. POC (10%), glycerol (30%); 2. 10% mixture of zein in 85% ethanol and PEG400; 3. Two-layer film, first with SDD (10%), second with zein (10%).	- the film with pumpkin oilcake showed a high sensitivity to humidity and water; these properties were improved by laminating the film with a hydrophobic layer of zein; - the highest value for EB was obtained for the first film, which was followed by the two-layer film and then the zein film; - the double-layer film showed a tensile strength 3 times lower than the pumpkin oilcake film and 4 times lower than the zein film.	[149]
POC (10%), glycerol (30%), guar–xanthan gum (0.2%), essential oil of thyme (1%) or basil (2%).	- the addition of essential oils increased the antioxidant activity of the films; - the greatest antimicrobial activity was shown by the films with 2% thyme essential oil.	[150]
POC (10%), basil or thyme essential oil (30–50 mL).	- the essential oils can contribute positively to the sensory properties of packaged products, also extending their shelf life; - the film with basil essential oil showed antibacterial activity against Listeria monocytogenes and Bacillus cereus; - the thyme essential oil film showed antibacterial activity against all microorganisms studied (*Escherichia coli*, *Salmonella enteritidis*, *Listeria monocytogenes*, *Staphylococcus aureus* and *Bacillus cereus*).	[151]
POC (10%), glycerol (30%), peppermint essential oil (1%).	- the films were used to pack *Afus Ali* grapes at room temperature and refrigeration, in all tested samples, over a certain period of time; the content of dry matter, the content of phenols and flavonoid substances and the sugar content decreased as a result of spoilage grapes; - the application of lower storage temperatures and active coating (with *Mentha piperita* essential oil) had a positive effect on all reactions; - the antioxidant character of grapes can be improved and/or maintained by applying films. The uncovered sample stored at room temperature had the greatest decrease in antioxidant activity values; - at the end of the storage period, the highest phenolic content was observed in samples with oil kept at room temperature and at refrigeration temperature compared to untreated samples and samples covered with sawdust films; - the microbiological results obtained are in the following order: film with peppermint essential oil < film with oilcake < control.	[152]
Double-layer membrane, the first contains POC (10%) and glycerol (30%), the second zein (10%).	- the film showed an increase in TS value in the third week of storage. The EB value decreased throughout the whole storage time (3 times lower than in the first week), hence the brittle/brittle film; - during the 4 weeks of storage, the film showed a slight decrease in humidity; the film showed good barrier properties against oxygen and moderate barrier properties for carbon dioxide in the first week; then, they both increased. A high presence of carbon dioxide has an important role in preventing microbiological contamination of products; - the film with scrap showed the oxygen barrier properties similar to a commercial packaging (made of polyamide-polyethylene). At the same time, it has a higher permeability for carbon dioxide, which in some cases can be beneficial: for example, the packaging of products with a high respiratory rate.	[153,154]
POC (10%), glycerol (0.25 g).	- the film with the best permeability and mechanical properties was obtained at pH 12 and a temperature of 90 °C; - regarding the antioxidant activity, the best values were obtained for the film prepared at pH 10 and a temperature of 60 °C; - the moisture content was not significantly affected by pH and heating temperature; - the films are strong and elastic, with good gas barrier properties; together with acceptable physical integrity, the EB value is similar or higher than that of cellophane.	[133]
**RAPESEED OILCAKE (ROC)**		
Protein hydrolysates from ROC with different degrees of hydrolysis—3, 6, 9, 12% (in proportion of 2%), chitosan (2%), glycerol (20%).	- the addition of protein hydrolysates increased the antibacterial ability; the best was observed for a degree of hydrolyzation of 12%; - increasing the degree of hydrolysis increased the compatibility with chitosan and implicitly increased the product’s mechanical properties and barrier against water.	[155]
ROC (60%), glycerol (40%), polycaprolactone (0–20%).	- the temperature increase in the injection molding process led to an increase in the viscoelastic properties and a decrease in the water absorption capacity; - the processing of the sawdust (by pelletizing, sieving and sorting) produced an increase in the viscoelastic modulus and stretching properties as well as a homogeneous and dense structure.	[156]
Protein isolates from ROC (5–7.5%), glycerol (30–50%).	- increasing the glycerol concentration of the strong, easily malleable, transparent and high VP film product; - increasing the protein concentration of the fragile, non-malleable, opaque and high WVP film product.	[157]
Protein from ROC (3 g), gelatin (3 g), sorbitol (2 g), sucrose (0.5 g), polysorbate 20 (1.5%), grapefruit seed extract (0–1.5%).	- the addition of grapefruit seed extract to films inhibited the growth of pathogenic bacteria such as *Escherichia coli O157:H7* and *Listeria monocytogenes*; - the packaging of Machyang strawberries with films with the addition of 1% extract decreased the population of aerobic bacteria, yeasts and molds after 14 days compared to the control sample; - the addition of the extract produced higher scores in the sensory analysis compared to the control sample.	[158]
Proteins from ROC (5%), glycerol (50%), sorbitol (50%), polyethylene glycol 400 (50%), genipin (0–10%).	- as the plasticizer changed from sorbitol to PEG-400 to glycerol, the films became more flexible and permeable to water vapor; - when genipin was applied, the films became stronger, less malleable and more opaque.	[159]
Protein extracts from ROC (2–6%), sorbitol (1.5–2%), sucrose (0.5–1%), polysorbate 20 (0.5–2%), gelatin (2–5%), Gelidium corneum (0.5–1.5%).	- the values for TS and EB have been created; the films with a content of 3% protein and 3% gelatin showed the most expected mechanical properties.	[160]
Protein isolates from ROC (35%), sodium dodecyl sulfate (1–5%, DSS), sodium dodecyl benzene (1–5%, DBS), glycerol (15%), polyvinylpyrrolidone (2%), zinc sulfate (1%).	- the functional properties of protein isolates are affected by DSS and DBS; - DBS was more effective than DSS in denaturing protein molecules, which led to increased TS and hardness; - the water absorption capacity of the protein isolates was improved with DBS; instead, DSS decreased the capacity; - both treatments increased the fat absorption capacity and suppression of emulsification activity.	[161]
Two membranes were made: 1. Proteins extracted from ROC (3–6%), Glidium corneum powder (arrowhead containing red algae 0.5–1.5%) sorbitol (1.5–2%), sucrose (0.5–1%), polysorbate 20 (1.5%) 2. Proteins extracted from ROC (3–6%), gelatin (2–5%), sorbitol (1.5–2%), sucrose (0.5–1%), polysorbate 20 (1.5%).	- the addition of Glidium corneum powder or gelatin created the physical properties of the film; - among all the formulations, the film containing 3% protein isolates, 3% gelatin, 2% sorbitol, 0.5% sucrose and 1.5% polysorbate 20 presented the most expected mechanical properties; - increasing the concentration of powder, protein isolates and gelatin also led to an increase in WVP; - the films with gelatin presented a denser and thicker structure than those with Glidium corneum. Instead, both have surface cracks due to protein isolate crystals.	[160]
**SUNFLOWER OILCAKE (SFOC)**		
SFOC (10%), glycerol (10%).	- the final products were firm, smooth, flexible, dark green–brown, and shiny, with a specific smell of sunflower; - with the increase in temperature and pH, the tensile strength increased. The highest EB value was obtained for films with pH 12 and 60 °C; - the WVP and solubility were uniform but decreased at high temperatures; - the optimal films were obtained at pH 12 and 90 °C.	[162]
SFOC, glycerol (30%), guar–xanthan (0.2%), essential oil of parsley and rosemary (0.25–1%).	- the TS values decrease in those related to EB and the antioxidant activity increases; - WVP decreases with the addition of parsley essential oil and increases with the addition of rosemary essential oil; - increasing oil concentration produces more fragile and elastic films.	[163]
SFOC protein isolates or soy protein isolates (5%), glycerol (1.5%), bovine blood plasma protein hydrolysates (HPSB, 10–40%).	- HPSB conferred antioxidant properties; the films showed an increase of about 64% by adding 40% HPSB; - HPSB caused a decrease in tensile strength, modulus of elasticity and glass transition temperature as well as an increase in elongation at break and water vapor permeability without visibly affecting the appearance of the films; - the hydrolysates had a plasticizing effect on the films.	[164]
SFOC (0.1–0.5 g), glycerol (0.5 g), sodium alginate (1 g), water (100 mL).	- the thickness, a_w_, time of solubility, oil and oxygen permeability increased with the addition of oilcake while moisture, WVP and solubility decreased; - the films exhibited high absorption of UV radiation and microbial stability, so they can be consumed together with the food packaged; - the films are suitable for the packaging of a wide range of foods (powdery products, food susceptible to oxidation, sliced dairy and meat products).	[165]
**PEANUT OILCAKE (PEOC)**		
Protein extracts from PEOC (8–14%), glycerol (0–15%)/citric acid (0–3%).	- increasing glycerol concentration decreased tensile strength and increased elongation at break; - the addition of citric acid as a crosslinking agent improved the mechanical properties and thermal resistance without affecting the water vapor permeability.	[166]
Pea starch (0–5 g), PEOC protein isolates (1–5 g), glycerol (1.5 g).	- EB increased and TS decreased with the incorporation of protein isolates and starch at a level of 50% (2.5 g), indicating that they could be found to considerably increase the flexibility of the film; - when protein isolates and starch were added at the level of 40% (3 g and 2 g), the water vapor permeability decreased significantly.	[167]
PEOC defatted flour (4%), glycerol (25%).	- improves coloration and elongation at break and decreases tensile strength and water permeability; - the films present adequate physico-chemical, optical, barrier and mechanical properties; - the film is able to improve the chemical stability of sunflower oil stored for 67 days at room temperature by preventing lipid oxidation.	[168]
Glycosylated PEOC protein isolates with xylose (5%), glycerol (15–45%).	- increasing the concentration of glycerol decreased the tensile strength and increased the affinity for water and the elongation resistance; - the films produced by dissolving the powder at 20 °C and adding 20% glycerol had mechanical properties and a water affinity comparable to the other vegetable protein films.	[169]
Protein isolate from PEOC (8%), glycerol (15%), Tween 80 (0.2%), thymol (0–2%).	- incorporation of thymol led to a decrease in transparency, WVP, TS and EB; - antimicrobial (against *Staphylococcus aureus*, *Lactobacillus plantarum*, *Escherichia coli*, *Pseudomonas aeruginosa*) and antioxidant activity increased significantly; - the highest antioxidant activity was found in the film with 2% thymol; the values were 5.29 times higher than those in the control film; - at low concentrations of thymol, the mechanical properties were not affected. Over 1% of values for TS and EB decreased.	[170]
**SESAME OILCAKE (SOC)**		
Proteins extracted from SOC (5%), glycerol (40%), 2 types of nano-clays: Cloisite 10 A, Cloisite Na^+^ (1–7%).	- the addition of nano-clays improves the mechanical properties and WVP; - studies must be carried out on the safety of using nanogels in the development of films for the packaging of food products and their toxic effect; - concentrations greater than 3% Cloisite 10 A produced a greater decrease than Cloisite Na^+^ in WVP values. The decrease was obtained when the content of nano-clays was 7%; - the addition of nano-clays increased the TS of the films. The highest value was obtained for the film with 5% Cloisite Na^+^.	[171]
Double layer edible coating: 1. Protein isolates from SOC crosslinked with 5% citric, malic and succinic acid (300 mL), glycerol (10%), 0.5% guar gum (200 mL); 2. Calcium chloride (2%) and pineapple juice extract (30%).	- the pineapple pieces were covered by the immersion method and can be stored in polystyrene trays for 15 days at 5–8 °C; - all coatings improve the durability and nutritional quality of fresh pineapple for 15 days; - the films with crosslinked proteins were more effective than those without crosslinking aid; - all coated samples showed a lower degradation of amino acids, carotenoids and phenolic compounds compared to uncoated samples; - scores for color, smell, texture, taste and overall acceptability were higher in coated samples.	[172]
Protein isolates from SOC (3%), glycerol (40%). The film-forming solution and formed films were subjected to ultraviolet (UV) radiation in 3 different regions: A (315–400 nm), B (280–315 nm) and C (200–280 nm).	- by applying UV treatments, films with a more compact structure without holes or cracks were obtained; - moisture, solubility and WVP decreased and density increased; - the mechanical properties were created; the highest values for TS and EB were obtained when the film-forming solution was subjected to UV-C treatment; - the UV treatments were more effective for creating properties on forming solutions than on formed films. Of all the treatments, those with UV-C were the most effective; - the UV treatment changed the intermolecular interactions, leading to an increase in the crystallinity and physical properties of the films.	[173]
Protein isolates from SOC (3%), glycerol (40%), titanium dioxide nanoparticles (TiO_2_, 1–5%).	- by adding 3% TiO_2_ the water vapor transmission rate and solubility decreased and the TS and opacity increased. Adverse effects on mechanical and water barrier properties were observed when the content increased to 5%; - films with 5% TiO2 are able to capture 10.96% of oxygen from the atmosphere over a period of 6 h.	[174]
Protein isolates from SOC (3–9%), glycerol (10–50%).	- the response surface methodology was used to optimize the parameters (pH, temperature, proteins, plasticizer) for the preparation of the edible film; - optimized values indicated that edible films prepared with 9% protein at pH 12, temperature 90 ºC and 10% plasticizer led to lower WVP values and maximum TS and solubility values; - the films with 7.2 g protein isolate and 1.8 g gum (ratio 80:20) showed the highest values for TS and the lowest for WVP and solubility. At the same time, they presented the best thermal and morphological properties.	[175]
Protein isolates from SOC (4.5–9 g), rosin gum (0–4.5 g), glycerol (10%).	- the addition of rosin gum to SDS protein isolate films increased moisture resistance, WVP, optical, mechanical, thermal (higher glass transition temperature values compared to films from other sources indicating stronger films) and morphological (compact, less porous and rough) properties; - the films with 7.2 g protein isolates and 1.8 g gum (ratio 80:20) showed the highest values for TS and the lowest values for WVP and solubility. At the same time, they presented the best thermal and morphological properties.	[176]
**WALNUT OILCAKE (WOC)**		
WOC (6%), glycerol (10%).	- protective coating against lipid damage and preserves sensory properties of coated nuts; - nuts coated with SDN flour were appreciated by consumers more than those coated with carboxymethylcellulose; - on day 84, the coated walnuts showed the highest values for carotenoids and tocopherols and the lowest values for the ratio between oleic and linoleic acids; - SDN is a residue from the nut oil industry and has a low cost.	[177]
WOC (6%), glycerol (10%), polyphenols extracted from WOC (0.5 mL).	- the walnut core was subjected to three treatments: the first without the addition of polyphenols (control), the second with the addition of polyphenols and the last with the addition of BHT; - on the last day of storage, the samples with the addition of polyphenols compared to the control sample showed the lowest values for the peroxide index, the content of anisidine and conjugated dienes; - on the last day, the control sample showed the greatest deterioration of polyunsaturated fatty acids, carotenoids and tocopherols; - the sample with polyphenols presented the highest acceptability score (6.72 on the hedonic scale of 9 points).	[178]
**LINSEED OILCAKE (LSOC)**		
Protein hydrolysates from LSOC (0–0.60%), alginate (1.5%), glycerin (0.6 g).	- the incorporation of protein hydrolysates did not affect the moisture content, solubility and barrier properties against oils, but it increased the thickness and WVP; - increasing the addition of hydrolysates to the product led to a decrease in light permeability; the film became darker, with a more yellowish and reddish tint; - the total content of polyphenols, antioxidant and antimicrobial activity increased, the films showed an inhibitory effect against *Staphylococcus aureus*, *Colletotrichum gloeosporioides* and *Rhizopus oligosporus* but not against *Escherichia coli*; - in the migration tests, the films released more than 60% of the active peptides within 30 min; - began to promise that the films are like active packaging for the preservation of fatty foods susceptible to oxidation.	[179]
**CHIA OILCAKE (COC)**		
Mucilage extracted from COC (1.5%), glycerol (35%), Tween 20 (15%).	- the films presented good mechanical and barrier properties. WVP values were greater than LDPE; - films with mucilage were more elastic and thicker than those made with whole seeds; - in appearance, the films were slightly reddish/yellowish but still transparent (good visible light barrier).	[180]
Mucilage extracted from COC (1.5%), glycerol (35%), Tween 20 (15%), essential oils (oregano and savory, 0–1.5%).	- the essential oils affected the moisture, solubility, optical and antimicrobial (the addition of an amount ≥1% inhibited mold growth between 38.01% and 77.66%) properties of the films; - the films displayed a homogeneous surface without pores or cracks, the addition of essential oils led to modification in the mechanical properties (significant decrease in TS and EB) and film microstructure (rough and heterogenous surface, more visible in the films with savory essential oil than in oregano).	[181]

## 8. Challenges and Future Directions

The research area of edible packaging is continuously evolving (new raw materials, active packaging development, nanotechnology applications) in an effort to develop films with properties similar to conventional synthetic polymers. The research carried out so far highlights two different directions for the valorization of oilcakes. The first includes the possibility of adding appropriate amounts as functional ingredients in food products (bakery and dairy) without negatively influencing the quality of the final products. The second consists of the extraction of protein for the development of edible films and coatings. Additionally, there are limited studies on the total incorporation of these by-products into edible packaging.

## 9. Conclusions

The food packaging sector is in constant development and changing, the natural and sustainable materials being the most researched field. Edible packaging is a sustainable and biodegradable alternative in the food packaging field because it provides quality optimization and waste reduction.

The edible packaging materials are promising due to their potential to be made from a variety of materials or to be carriers to active substances (antimicrobials and/or antioxidant agents). The materials used derivate from edible ingredients and can be consumed by humans without health risks.

The agro-industrial residues are a valuable source of bioactive compounds that can be valorized and used in edible films and coatings because the bioactive compounds improve the functions and nutritional properties of the packaging.

The incorporation of oilcakes in edible packaging is a promising strategy to achieve sustainability, circular economy and the minimization of food waste and losses.

## Figures and Tables

**Table 1:** Study of the chemical compositions of edible oilcake obtained after cold pressing [14,15,16,17,18,19,20,21,22,23,24,25,26,27,28,29,30,31,32,33,34,35,36,37,38,39,40,41,42,43,44,45,46,47,48,49,50,51,52,53,54,55,56,57,58,59,60,61,62].

Oilcake	Moisture, %	Proteins, %	Lipids, %	Ash, %	Fibers, %	Carbohydrates, %	Energy Value, kcal/100 g
Sunflower	2.50–11.00	19.93–44.90	7.00–16.60	4.56–8.00	17.40–36.52	15–28.2	237.52–514.84
Pumpkin	5.00–8.20	29.39–53.98	5.92–36.22	4.20–8.70	3.89–7.10	15.88–19,73	242.14–635.02
Rapeseed	6.00–10.80	14.03–40.10	5.14–23.10	5.00–19.70	5.50–15.46	25.1–48	213.78–591.22
Sesame	1.17–16.80	16.96–45.90	5.10–48.00	3.80–12.40	3.28–22.70	22.5–46.96	210.3–848.84
Flaxseed	6.89–9.27	14.40–41.97	6.11–21.40	4.70–6.27	6.29–12.90	16.26–52.45	190.21–596.08
Hemp	6.35–13.61	23.25–33.45	0.51–14.02	3.30–9.78	17.41–60.38	2.80–48.54	143.61–574.90
Chia	6.80–10.84	28.20–35.00	6.52–11.39	4.58–6.27	23.81–30.24	23.53–30.24	313.22–423.95
Soybean	8.40–9.66	43.30–45.50	9.30–15.55	5.71–5.91	4.95–11.28	14.98–21.76	326.72–431.55
Walnut	3.60–10.50	10.30–50.40	7.95–36.80	2.79–10.00	6.79–18.50	17.4–49.75	195.93–768.80

The carbohydrates content was calculated by difference (100 − (proteins + fat + ash + fiber + moisture)), while the energy values were calculated by multiplying nutrient values with the conversion coefficients (4 for proteins and carbohydrates, 9 for lipids and 2 for fibers) [63].

## Data Availability

The data presented in this study are available on request from the corresponding author.
